# Rab7 Associates with Early Endosomes to Mediate Sorting and Transport of Semliki Forest Virus to Late Endosomes

**DOI:** 10.1371/journal.pbio.0030233

**Published:** 2005-06-21

**Authors:** Andreas Vonderheit, Ari Helenius

**Affiliations:** **1**Institute of Biochemistry, Swiss Federal Institute of Technology, Zurich, Switzerland; Max-Planck-Institute of Molecular Cell Biology and GeneticsGermany

## Abstract

Semliki forest virus (SFV) is internalized by clathrin-mediated endocytosis, and transported via early endosomes to late endosomes and lysosomes. The intracellular pathway taken by individual fluorescently labeled SFV particles was followed using immunofluorescence in untransfected cells, and by video-enhanced, triple-color fluorescence microscopy in live cells transfected with GFP- and RFP-tagged Rab5, Rab7, Rab4, and Arf1. The viruses progressed from Rab5-positive early endosomes to a population of early endosomes (about 10% of total) that contained both Rab5 and Rab7. SFV were sequestered in the Rab7 domains, and they were sorted away from the early endosomes when these domains detached as separate transport carriers devoid of Rab5, Rab4, EEA1, Arf1, and transferrin. The process was independent of Arf1 and the acidic pH in early endosomes. Nocodazole treatment showed that the release of transport carriers was assisted by microtubules. Expression of constitutively inactive Rab7T22N resulted in accumulation of SFV in early endosomes. We concluded that Rab7 is recruited to early endosomes, where it forms distinct domains that mediate cargo sorting as well as the formation of late-endosome-targeted transport vesicles.

## Abstract 2

Using fluorescently tagged Semliki Forest Virus (SFV) as cargo, the authors observed trafficking of the virus through the endocytic pathway and uncovered new details about early-to- late-endocytic vesicle transitions.

## Introduction

In the classical clathrin-mediated endocytic pathway, the step from early to late endosomes is crucial for selective transport of cargo and membrane components to lysosomes for degradation. This step involves cargo sorting and segregation, and the transfer occurs either by carrier vesicles that detach from early endosomes, or by the maturation of early endosomes to late endosomes [1–5].

Whereas vesicle traffic from the plasma membrane to early endosomes and homotypic fusion of early endosomes is regulated by a small GTPase, Rab5 [6,7], and recycling to the plasma membrane depends on Rab4 and Rab11 [8,9], the factors that mediate cargo transfer from early to late endosomes remain largely unidentified. In vitro experiments with biochemically purified endosomes have suggested that this step is mediated by a GTPase, Arf1. Together with COP I, it is thought to drive the formation of endosomal carrier vesicles (ECVs) [10–13]. Low pH inside the endosomes was found to be essential for this process.

There is also evidence that Rab7 is critically involved in early to late endosome traffic. Expression of dominant negative Rab7 mutants blocks the exit of certain cargo molecules from early endosomes to late endosomes [14–16]. However, since Rab7 has been localized to late endosomes and lysosomes, it has been proposed that it is not involved in the formation of carrier vesicles at the early endosome but in downstream events [17]. That Rab7 is also involved in a later step—in trafficking from the late endosome to the lysosome—is supported by observations in many systems [18–21].

In this study, we have analyzed the sorting of cargo from early to late endosomes using triple-colored, video-enhanced fluorescence microscopy in live cells expressing various Rab- and Arf-GTPases labeled with green, red, yellow, or cyan fluorescent protein (GFP, RFP, YFP or CFP). As endocytic cargo, we used fluorescently labeled Semliki forest virus (SFV)—a simple, enveloped RNA virus known to enter cells via the classical clathrin-mediated pathway and to be degraded in lysosomes [22–24]. The main advantage of SFV over most physiological ligands is that fluorescently labeled, individual particles can be visualized and tracked during entry as single identifiable fluorescent spots. Our results show that the transport of SFV occurs in Rab7-positive vesicles formed from Rab7-positive domains in early endosomes. Although present in early endosomes, Arf1 did not appear to be involved.

## Results

### Characterization of Labeled SFV

**Figure 1:**
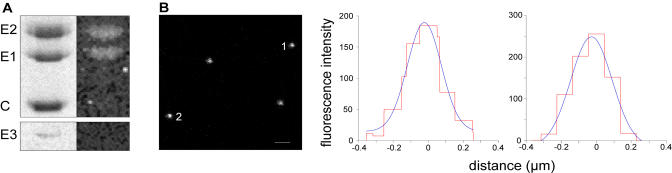
Fluorophores Label Glycoproteins E1 and E2 of SFV (A) Analysis of SFV-FITC by non-reducing SDS-PAGE with Coomassie blue staining in the left lane and fluorography in the right lane. E1 (49 kDa) and E2 (52 kDa) are the only proteins that are fluorescently labeled; capsid protein C and E3 are not. (B) Confocal microscopy of Cy5-labeled SFV, showing individual spots of uniform size (approximately 0.4 μm). The relative fluorescence intensity distribution of two spots is shown. The distribution of the fluorescence fits to a Gaussian function. (Fit is calculated using the equation: *y* = *e* ^−*x*^2^.) Scale bar represents 1 μm.M^

To visualize the entry of SFV in live cells by fluorescence microscopy, we labeled purified virus with different fluorophores: Cy5 (SFV-Cy5), AlexaFluor594 (SFV-AF594), and FITC (SFV-FITC). SDS-PAGE and fluorography of the labeled viruses showed that the fluorophores were coupled to glycoproteins E1 and E2 (shown for SFV-FITC in Figure 1A). Together with the small glycopeptide E3, E1 and E2 form interconnected spike complexes in the envelope [25]. As expected from its location on the lumenal side of the viral envelope, the capsid protein C was not labeled. Absorbance spectra indicated that virions carried an average of 400 molecules of dye. The labeling process reduced infectivity by about half, from 7 × 10^7^ to 3 × 10^7^ plaque forming units (PFU) per microgram of viral protein.

When viewed by fluorescence microscopy, the fluorescent virus particles were visible as spots with a diameter of 0.4–0.5 μm (shown for SFV-Cy5 in Figure 1B). The distribution of the signal could be fit to a single Gaussian function (blue line in Figure 1B) with *R* ^2^ = 0.96, suggesting that the light was emitted from single particles. Structural studies have shown that the actual diameter of a SFV particle is 70 nm [25]. When the labeled SFV were allowed to bind to the surface of Vero cells, the staining of the fluorophore-labeled viruses overlapped completely with the pattern observed by immunofluorescence using a polyclonal antibody against E1 and E2 (Figure S1).

### Internalization, Penetration, and Degradation

SFV particles were bound to the cell surface at 4 °C for 1 h at a multiplicity of infection (MOI) of 20, the unbound virus was washed away, and the cells were shifted to 37 °C. Under these conditions, the viruses are rapidly internalized by clathrin-coated vesicles and delivered to endosomes and subsequently to lysosomes, where proteolytic degradation of envelope proteins and unfused viruses occurs [22].

**Figure 2:**
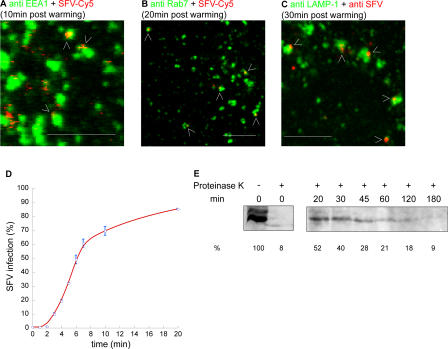
Internalization, Penetration, and Degradation SFV-Cy5 (at a MOI of 20) was allowed to bind to Vero cells for 60 min at 4 °C, followed by transfer to 37 °C for different time periods. (A) The cells were fixed and permeabilized 10 min after warming, followed by immunostaining using antibodies to EEA1. SFV-Cy5 is present in EEA1-positive endosomes. (B) The cells were fixed 20 min after warming and immunostained with anti-Rab7 antibodies. SFV-Cy5 is present in Rab7-positive endosomes. (C) Non-labeled SFV was used, and the cells were fixed and permeabilized 30 min after warming and immunostained with anti-LAMP-1 and anti-E1/E2 antibodies. SFV is present in endosomes positive for LAMP-1. (D) A FACS-based infection assay was used to determine the time course of SFV penetration during entry. Vero cells were infected with a MOI of one. NH_4_Cl was added at different time points to inhibit infection. Cells were further incubated at 37 °C for 5 h, then fixed and immunostained for newly synthesized glycoproteins (*n* = 3). (E) Analysis of E1/E2 degradation was determined using immunoblotting. Virus (MOI of 50) was bound to Vero cells in the cold, and unbound virus was washed away. Cells were incubated for indicated times, and surface-associated viruses were removed by Proteinase K treatment. Cells were lysed, and after SDS-PAGE, immunoblotting was performed with an antibody against E1/E2. Note that contrary to non-reduced samples (Figure 1A), E1 and E2 co-migrate in SDS-PAGE after reduction. Scale bars represent 5 μm.

The progressive movement could be followed using the fluorescently labeled virus in Vero cells (Figure 2A–2C). As previously reported [26], the viruses were first observed as individual spots on the cell surface. Confocal microscopy of fixed and immunostained cells 10 min after warming showed the majority of internalized viruses in compartments positive for early endosome antigen 1 (EEA1), a Rab5 effector and an early endosome marker [27,28] (Figure 2A). At this time, many virus particles were still on the cell surface (Figure S2). After 20 min of warming, many viruses were already present in compartments positive for Rab7 (Figure 2B), a late endosomal marker [29]. Only after 30 min or longer could overlap be observed with lysosome associated membrane protein 1 (LAMP-1), a marker for lysosomes [30,31] (Figure 2C).

To determine the timing of the acid-activated penetration event leading to infection, SFV was allowed to bind to the cells in the cold at a MOI of one. At different times after warming, 20 mM NH_4_Cl was added. Like other lysosomotropic weak bases, NH_4_Cl raises the pH in acidic organelles almost instantaneously [32], and prevents further acid activation of incoming viruses. After 5 h, the fraction of infected cells was determined using an indirect FACS-based assay, in which newly synthesized viral proteins were detected with an anti-E1/E2 antibody. In agreement with results from other cell types [33], the acid-induced fusion events started between 2 and 3 min after warming and reached a half maximal level at 6 min (Figure 2D).

Following a lag phase, SFV endocytosis is known to result in efficient degradation of E1 and E2 proteins in lysosomes [34]. When degradation was analyzed in Vero cells by immunoblotting using anti-E1/E2 antibodies, it was found to start 30 min after warming (Figure 2E). In this experiment, Proteinase K–mediated removal of surface-bound viruses showed that about half of the cell-associated virus particles (52%) were endocytosed.

For the incoming virus, the course of events in Vero cells thus followed a program that involved (1) rapid internalization, (2) exposure to low pH in early endosomes (2–15 min), and (3) transfer to late endosomes and lysosomes (starting after 20 min). Through all these different compartments, the size and intensity of fluorescent spots representing individual virus particles remained roughly unaltered (data not shown)**.** This meant that even when the viruses fused and E1 and E2 became part of the endosomal membrane, the glycoproteins did not diffuse away from each other. This behavior of SFV membranes has previously been observed after fusion with the plasma membrane [35].

### Localization of Endocytic Markers

**Figure 3:**
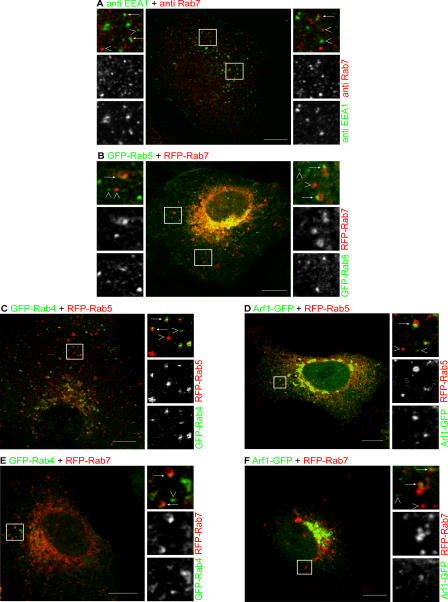
Endosomes Labeled with Two Markers Show Presence of Rab7 in Early Endosomes Vero cells were fixed and viewed by confocal microscopy. (A) Confocal microscopy of immunolabeled cells using anti-EEA1 (green) and anti-Rab7 (red) antibodies, (B–F) The cells were transfected with fluorescent-protein-labeled constructs as follows: (B) GFP-Rab5 and RFP-Rab7, (C) GFP-Rab4 and RFP-Rab5, (D) Arf1-GFP and RFP-Rab5, (E) GFP-Rab4 and RFP-Rab7, and (F) Arf1-GFP and RFP-Rab7. Arrowheads show individual endosomes positive for one of the two markers, and arrows indicate endosomes positive for two markers. Scale bars represent 10 μm.

To study the distribution of markers in endosomal compartments, we performed double immunofluorescence experiments with untransfected cells. We used antibodies to EEA1 (an effector of Rab5) [27,28], and antibodies to Rab7 [36]. When the distribution was compared, we observed, as have others, that these early and late endosomal markers were largely separated (Figure 3A, arrowheads). However, closer inspection and quantification showed that 13.6% of the EEA1-positive organelles were also stained with anti-Rab7 antibodies (arrows). In CV-1 and HeLa cells the corresponding numbers were 13.1% and 12.8%, respectively (Figures S3 and S4).

To determine whether the overlap was coincidental, we quantified the extent of overlap between EEA1 and COP II, which do not associate with common organelles (Figure S5). The apparent overlap was 4.6% (Figure S5B and S5C). The measured overlap between EEA1 and caveolin-1, which is known to form a domain in some of the early endosomes [37], was 10.5% (Figure S5A). We concluded that the co-localization of EEA1 and Rab7 was real, and that about one in ten EEA1-positive endosomes contained detectable amounts of Rab7. The double-labeled endosomes often displayed distinct red and green regions, suggesting location of EEA1 and Rab7 in separate domains of the mosaic structure of the endosomal membrane (Figures 3A, S3, and S4, arrows) [38,39].

To analyze the endosomes in live cells, we expressed a variety of fluorescent-protein-labeled small GTPases known to occur in distinct endosomal compartments: Rab5 for early endosomes, Rab4 for early and recycling endosomes, and Rab7 for late endosomes [6,8,29]. We also expressed Arf1, which has been reported to be present on early endosomes and ECVs [12].

First, the localization of GFP-Rab5 and RFP-Rab7 was determined in fixed, transfected cells by confocal microscopy in the absence of virus (Figure 3B). As expected, most of the Rab5- and Rab7-positive structures were localized in distinct organelles, at least when viewed in the peripheral regions of the cytoplasm where individual organelles were easily distinguished (arrowheads). However, again in every cell a population of GFP-Rab5-positive endosomes (14.6%) could be observed also positive for RFP-Rab7 (arrows). That these were indeed hybrid organelles was confirmed in live video recordings of unfixed cells by the coordinated movement of both fluorescent colors (Videos S1–S3). The distinct red and green regions (domains) in the endosomes were persistent features also in live cells.

When GFP-Rab4 and RFP-Rab5 were co-expressed, 37.7% of the Rab5-positive endosomes contained Rab4, and when Arf1-GFP and RFP-Rab5 were expressed, 35.5% of the Rab5 endosomes contained the labeled Arf1 (Figure 3C and 3D), confirming that early endosomes are heterogeneous and contain different small GTPases. When RFP-Rab7 and GFP-Rab4 were co-expressed, some double-stained vesicles (12.3%) could also be observed (Figure 3E, arrows). The same was observed for Arf1-GFP and RFP-Rab7 (12.5%) (Figure 3F). These findings were consistent with the presence of Rab7 in some early endosomal structures. As expected, immunofluorescence showed that Rab7 was present in LAMP-1-positive structures. We found that of Rab7-positive organelles, 46% contained LAMP-1 (data not shown).

### SFV in Rab7-Positive Early Endosomes

**Figure 4:**
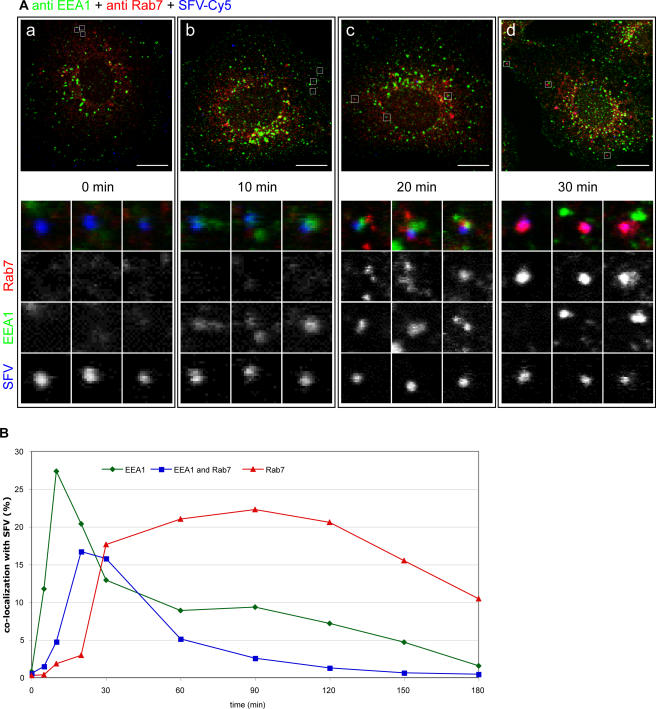
Progression of SFV through Endosomes Labeled for EEA1 and Rab7 (A) Confocal microscopy of Vero cells. SFV-Cy5 was allowed to bind to Vero cells (at a MOI of 20) for 60 min at 4 °C, followed by transfer to 37 °C and fixed after different times (a, 0 min; b, 10 min; c, 20 min; d, 30 min) and immunolabeled with EEA1 and anti-Rab7 antibodies. Shown are three viruses for each time point. Scale bars represent 10 μm. In panel a, SFV do not co-localize with any endosomal marker; in panel b, SFV are localized in EEA1-positive endosomes; in panel c, SFV are localized in EEA1- and Rab7-positive endosomes; and in panel d, SFV are localized in Rab7-positive endosomes. (B) Quantification of SFV-Cy5 in endosomes that contain EEA1 but no Rab7 (green line), endosomes that contain both (blue line), and structures that contain only Rab7 (red line) over time. The values are normalized such that 100% represents the number of cell-associated viruses at each time point corrected for the degradation.

To analyze the endosomes that were reached by the virus, SFV-Cy5 was allowed to bind to Vero cells in the cold. The particle to cell ratio was kept low (20 PFU/cell) so that individual particles could be followed, and endosomes would not receive multiple SFV particles. The cells were then washed, shifted to 37 °C for different times up to 180 min, fixed, immunolabeled with anti-EEA1 and anti-Rab7, and analyzed by confocal microscopy. As shown in Figure 4A (panel a), at 0 min the SFV particles (blue) did not co-localize with either of the endosomal markers (red and green). After 10 min (Figure 4A, panel b), most of the virus particles co-localized with EEA1 in early endosomes. After 20 min (Figure 4A, panel c), many of the virus particles were located in the endosomes positive for both EEA1 and Rab7, and from 30 min onwards (Figure 4A, panel d), an increasing fraction was seen in organelles stained by Rab7 alone.

To quantify these observations, we viewed 260 virus particles in more than ten cells for each time point, and scored how many co-localized with different endosomal markers. The results (Figure 4B) were normalized so that 100% represented the number of cell-associated viruses at each time point corrected for the degradation. The results showed a progression of the internalized viruses through different endosome subpopulations. Co-localization with EEA1 without Rab7 peaked at 10 min (Figure 4B, green line). It dropped off rapidly and was replaced by a population positive for both EEA1 and Rab7 (blue line), with a maximum at 20 min. This was followed by organelles positive for Rab7 but negative for EEA1 (red line) starting at 30 min and persisting until 180 min.

What was new and unexpected in these observations was that the viruses passed through an endosomal organelle population that contained both EEA1 and Rab7. This occurred in the time period 10–60 min after warming. As shown above, such EEA1- and Rab7-positive organelles were present whether SFV was added or not.

We modeled SFV transit through the different endosomal compartments using coupled first-order reactions (Kinetikit/GENESIS) [40] assuming unidirectional transport: [plasma membrane] → [early EEA1-positive endosome] → [hybrid EEA1- and Rab7-positive endosome] → [late Rab7-positive endosome] → [degradation]. A set of parameters was found that allowed a fit with the experimental data in Figure 4B (Figure S6). These parameters suggested that all the viruses that ended up in late endosomes passed through the hybrid compartment. Moreover, the model allowed us to estimate the average residence times of SFV in the different endosomal compartments. They were 10 min for the early endosomal compartments, 16 min for the hybrid endosomes, and 52 min for the late endosomal compartments.

**Figure 5:**
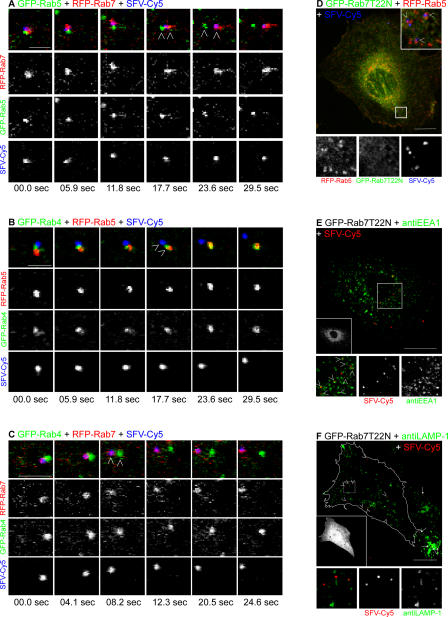
Sorting of Rab7 and SFV from Early Endosomes (A–C) Selected images obtained from a time series starting 20 min after warming. Vero cells were transfected with (A) GFP-Rab5 and RFP-Rab7, (B) GFP-Rab4 and RFP-Rab5, or (C) GFP-Rab4 and RFP-Rab7; SFV-Cy5 was then added. SFV-Cy5 is sorted away from Rab5- and Rab4-positive endosomes in a Rab7-positive vesicle (arrowheads). Scale bars represent 2.5 μm. (D) Confocal microscopy of a Vero cell transfected with the dominant negative GFP-Rab7T22N and RFP-Rab5, followed by incubation with SFV-Cy5. Virus was internalized for 1 h at 37 °C. SFV-Cy5 is present in large Rab5-positive endosomes (arrowheads). Scale bar represents 10 μm. (E and F) Confocal microscopy of Vero cells transfected with the dominant negative GFP-Rab7T22N (insets), followed by incubation with SFV-Cy5. Virus was internalized for 1 h at 37 °C. Cells were fixed, permeabilized, and immunostained for (E) EEA1 or (F) LAMP-1. In all, 86.3% of SFV-Cy5 (red) is present in large EEA1-positive endosomes (green in [E]) and not in LAMP-1-positive structures (green in [F]) (arrowheads). Note that SFV-Cy5 is present in LAMP-1-positive structures in untransfected cells (arrows). Scale bars represent 10 μm.

When GFP-Rab5- and RFP-Rab7-transfected cells were allowed to internalize SFV-Cy5, the progression of the virus particles could be observed by confocal microscopy in live Vero cells (Figure 5A); the majority of viruses were first located in organelles that were positive for GFP-Rab5, later in organelles that were positive for both GFP-Rab5 and RFP-Rab7, and finally in RFP-Rab7- positive organelles. A similar progression of virus through endosomal compartments was observed in fixed and live CV-1 (Figure S7A and S7B) and HeLa cells (Figure S7C and S7D), although progress through the pathway was less synchronous in these cells. It is noteworthy that the expression of GFP-Rab5 and/or RFP-Rab7 did not affect SFV infection of Vero cells, consistent with reports that these chimeric proteins have no adverse effect on the pathway [37].

A closer look at the distribution of GFP-Rab5, RFP-Rab7, and SFV-Cy5 in endosomes showed a typical “mosaic” distribution of the Rabs [38]. GFP-Rab5 and RFP-Rab7 were as a rule distributed in distinct domains of the organelle (Figure 5A). That they were part of a common structure, and that the virus was trapped in it, was evident when endosomes in live cells were viewed over time; the three labels often moved together in the cytoplasm as parts of the same organelle (Videos S4 and S5). An uneven distribution of fluorescent markers was also seen in the endosomes of HeLa and CV-1 cells whether viewed after immunofluorescence staining for EEA1 and Rab7 in fixed, untransfected cells (Figure S7A and S7C), or viewed in live cells expressing the fluorescent-protein-labeled Rabs (Figure S7B and S7D).

### Sorting of Rab7 and SFV from Early Endosomes

When the fate of SFV-Cy5-containing endosomes was followed by video microscopy, separation of the RFP-Rab7-positive domain and the virus from the GFP-Rab5-labeled domains could frequently be seen. The hybrid endosome was split in two: one part contained the Rab5, and the other the Rab7 and the virus particle. In the examples shown in Figure 5A and Videos S4 and S5, the SFV-Cy5- and RFP-Rab7-positive particle moved away while the Rab5-containing remnant remained stationary. In 70% of the cases recorded (*n* = 23) this was the case. In the other 30% of cases, it seemed that both parts of the endosome moved away after fission, in opposite directions.

The viral cargo was thus transferred from an early endosome that contained both Rab5 and Rab7 to a detached organelle that contained Rab7 but no Rab5. In all analyzed time series, the virus always left the Rab5/Rab7 compartment together with Rab7, and Rab5 was left behind. We never observed the virus leaving an early endosome compartment without Rab7. It was therefore possible that formation of the Rab7-positive carrier vesicle was triggered by the presence of the virus. That the virus was not necessary, however, was shown by the observation that Rab7-positive vesicles could be seen leaving Rab5- and Rab7-positive endosomes in cells to which no virus was added (see Video S3).

To confirm that the organelles that SFV were leaving were in fact early endosomes, we transfected either RFP-Rab5 or RFP-Rab7 together with another early endosomal marker, GFP-Rab4. We then allowed SFV-Cy5 to be internalized for 20 min. Rab4 is known to be present in early and recycling endosomes [8,9]. In live recordings, the SFV glycoproteins (blue) could be observed to sort away from both Rab4 and Rab5 (Figure 5B; Video S6). In cells expressing GFP-Rab4 and RFP-Rab7 the departure occurred together with Rab7, leaving Rab4 quantitatively behind (Figure 5C; Video S7). Furthermore, we used fluorescently labeled transferrin. We loaded RFP-Rab5- or RFP-Rab7-expressing cells with transferrin-AlexaFluor488 and added SFV-Cy5 (Figure S8A and S8B; Videos S8 and S9). SFV was present in the transferrin- and Rab5-positive early endosomes. When the sorting of SFV and Rab7 took place 20 min after internalization, transferrin failed to be included in the carrier vesicles.

When a constitutively inactive GFP-Rab7T22N mutant and RFP-Rab5 were expressed together, we saw that the Rab5-positive early endosomes were bigger and more rounded than in cells without the Rab7 mutant (Figure 5D). The same was true for GFP-Rab7T22N-expressing cells stained for EEA1 (Figure 5E). Rab7 was not associated with any membrane organelles. When SFV-Cy5 was allowed to enter these cells for 1 h, we found that it was internalized but failed to leave the enlarged Rab5/EEA1-positive compartments. Consistent with capture in early endosomes, almost none of the SFV localized to LAMP-1 structures (Figure 5F, arrowheads), whereas in control cells many did (arrows). Quantification showed that 86.3% of the viruses remained co-localized with EEA1 and only 3.9% co-localized with LAMP-1-containing organelles. This indicated that forward transport of SFV was inhibited by GFP-Rab7T22N at the level of early endosomes [14–16]. Active Rab7 was evidently needed for the transfer of SFV from this compartment. The inhibition in transport to late endosomes was likely the reason why the early endosomes were enlarged in these cells.

### Sorting Is Arf1-Independent

**Figure 6:**
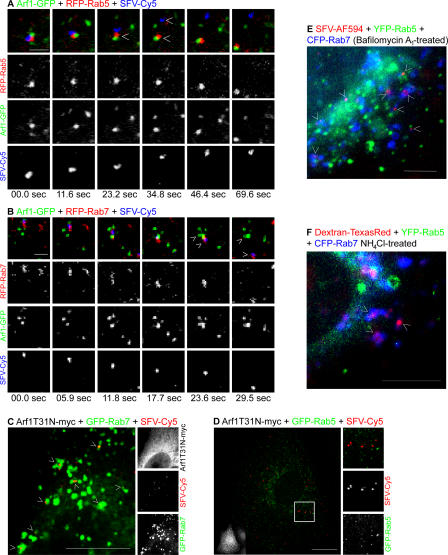
Sorting of SFV into Late Endosomes is Arf1- and pH-Independent (A and B) Selected images, obtained from time series recorded approximately 20 min after warming. Vero cells were transfected with (A) Arf1-GFP and RFP-Rab5, or (B) Arf1-GFP and RFP-Rab7, then SFV-Cy5 was added. Arf1 is present on Rab5-positive endosomes. SFV-Cy5 is sorted away from Arf1- and Rab5-positive endosomes in a Rab7-positive vesicle (arrowheads). Scale bars represent 2.5 μm. (C and D) Confocal microscopy of a Vero cell transfected with GFP-Rab7 (green in [E]) or GFP-Rab5 (green in [D]) and Arf1T31N-myc and incubated with SFV-Cy5 (red). Cells were fixed, permeabilized, and immunostained using an antibody against myc-tag (9E10) after 1 h of internalization. SFV-Cy5 is not present in Rab5 but is present in Rab7-positive endosomes (arrowheads), indicating that Arf1 is not needed for the sorting of SFV into Rab7-positive endosomes. Scale bars represent 10 μm. (E) Wide field fluorescence image of Bafilomycin A_1_–treated (25 nM) Vero cells expressing YFP-Rab5 and CFP-Rab7, at 2 h after internalization of SFV-AF594. SFV is present in Rab7-positive endosomes (arrowheads), indicating that no acidic pH is needed for the sorting of SFV into Rab7-positive endosomes. Scale bar represents 5 μm. (F) NH_4_Cl-treated (20 mM) Vero cells expressing YFP-Rab5 and CFP-Rab7 incubated with Dextran–Texas Red for 2 h. Dextran is present in Rab7-positive endosomes (arrowheads), indicating that no acidic pH is needed for the sorting of SFV and Dextran into Rab7-positive endosomes. Scale bar represents 5 μm.

Transport of cargo from early to late endosomes has been reported to occur in so-called ECVs regulated by Arf1 [12]. To test whether Arf1 mediates this sorting step for SFV, we transfected cells with Arf1-GFP and either RFP-Rab5 (Figure 6A; Video S10) or RFP-Rab7 (Figure 6B; Video S11). We then followed the fate of SFV-Cy5 in these cells over time in confocal video recordings.

It was relatively easy to find endosomes labeled with Arf1-GFP, RFP-Rab5, and SFV-Cy5. The three colors did not overlap completely, but moved together through the cytoplasm, indicating that Arf1, Rab5, and the virus were present in distinct domains of the same endosome. Figure 6A and Video S10 show how the virus leaves such an endosome. From several video movies like this, we concluded that when the virus leaves, both Arf1-GFP and RFP-Rab5 stay behind. A similar conclusion was reached when cells expressing RFP-Rab7 and Arf1-GFP were viewed. In this case, the virus and RFP-Rab7 left the endosome together, with Arf1-GFP staying behind (Figure 6B; Video S11). Furthermore, when we used the myc-tagged dominant negative mutant Arf1T31N, we found SFV-Cy5 in GFP-Rab7-positive endosomes and not in GFP-Rab5-positive endosomes after 1 h of warming (Figure 6C and 6D). Quantification showed that 86.6% of the viruses entered a Rab7-positive endosome, whereas only 5.9% remained in a Rab5-positive endosome. It was clear that SFV was not using Arf1-containing domains or vesicles for transport from the early to late endosomes, indicating that Arf1 was not needed for sorting of SFV into Rab7 vesicles.

It has been reported that the transport of certain cargo by ECVs from early to late endosomes is pH-dependent [12]. To test in live cells whether this is true for SFV, we used drugs (NH_4_Cl and Bafilomycin A_1_) to neutralize the pH in endocytic organelles. That these agents did indeed neutralize the pH as expected was shown by a 12-fold drop in SFV infectivity in a FACS-based assay (not shown).

We found that when SFV-Cy5 was allowed to enter for 2 h in the presence of the drugs, it reached Rab7-positive compartments devoid of Rab5 (Figure 6E). The same was observed for a fluid phase marker, Dextran–Texas Red (Figure 6F). We concluded that sorting of SFV from early endosomes to late endosomes did not require an acidic endosomal pH. Furthermore, since the virus did not penetrate under these conditions, sorting was independent of the fusion of the viral envelope with the endosomal membrane. Evidently, SFV was sorted into late endosomes and finally into lysosomes even if it had not released its capsid into the cytoplasm.

### The Role of Microtubules

**Figure 7:**
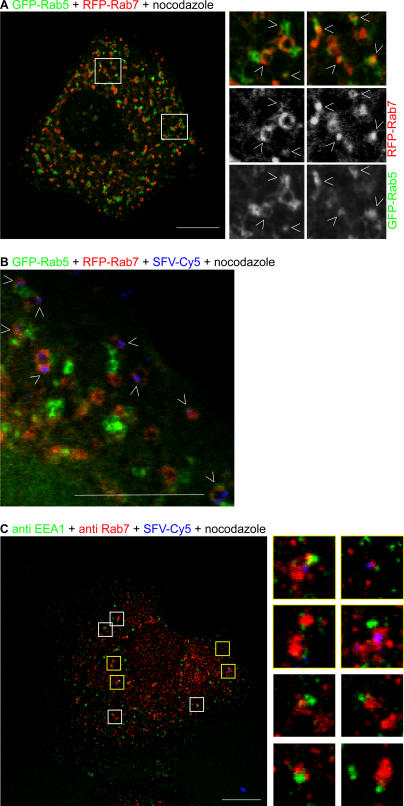
Endosomes Move along Microtubules (A) Confocal microscopy of a Vero cell transfected with GFP-Rab5 and RFP-Rab7 after treatment with 5 μM nocodazole. Endosomes are enlarged and many (approximately 75%) Rab5-positive endosomes are also positive for Rab7 (arrowheads). Scale bar represents 10 μm. (B) Cells treated as in (A) were incubated with SFV-Cy5, and fixed 1 h after warming. SFV-Cy5 is present in bigger endosomes positive for Rab5 and Rab7 (arrowheads). Scale bar represents 5 μm. (C) Confocal microscopy of a Vero cell treated with 5 μM nocodazole and incubated with SFV-Cy5. After 1 h of internalization at 37 °C, cells were fixed, permeabilized, and immunostained for EEA1 and Rab7. Endosomes positive for both markers can be seen (white rectangles) (41%), and SFV-Cy5 is captured in these structures (yellow rectangles). The protrusions of Rab5 shown in (A) are not seen in (C) and are probably caused by overexpression of the Rab proteins. Scale bar represents 10 μm.

It is well known that endosomes can move along microtubules [36,41–43]. When recording in cells expressing tubulin-GFP, complex bidirectional movement of GFP-Rab7-labeled late endosomes and RFP-Rab5-labeled early endosomes along microtubules could be monitored (Video S12). When the microtubule-disrupting drug nocodazole was added and the cells were incubated for 30 min, we observed larger and more numerous endosomes (Figure 7A). The majority of these were positive for both GFP-Rab5 and RFP-Rab7 (approximately 75%). Again the two Rabs were, as a rule, located in nonoverlapping domains (Figure 7A, arrowheads). In untransfected cells that were immunostained for EEA1 and Rab7 the effect of nocodazole was not as dramatic, but still many of the EEA1-positive endosomes were positive for Rab7 (approximately 41%) (Figure 7C).

When SFV-Cy5 was allowed to enter cells transfected with GFP-Rab5 and RFP-Rab7 and treated with nocodazole for 1 h, SFV-Cy5 was found to co-localize with the GFP-Rab5- and RFP-Rab7-positive endosomes (Figure 7B). We observed the same in untransfected cells immunostained for EEA1 and Rab7 (Figure 7C). These observations were consistent with a role for microtubules and microtubule-dependent motors in the separation of early endosomes and Rab7-positive carrier vesicles. That nocodazole had little effect on SFV infection (not shown) was expected given that penetration occurs at the level of early endosomes devoid of Rab7. Accordingly, expression of the dominant negative Rab5S34N mutant inhibits SFV infection whereas expression of the corresponding Rab7 mutant (Rab7T22N) does not [44].

## Discussion

The classical endocytosis pathway from clathrin-coated pits to lysosomes is well studied but far from fully understood. One of the unresolved issues concerns the mechanisms of molecular sorting in early endosomes, and the mechanism of cargo transfer from early to late endosomes and lysosomes. It is unclear whether the process involves maturation of early endosomes to late ones by gradual modification of membrane components, or by the formation of carrier vesicles between permanent compartments [45]. As reported by others the limiting membrane of endosomes contains a mosaic of domains with different composition and distinct functions [17,38,39,46,47]. In early endosomes, the Rab5 domains are the most prominent and best analyzed. Rab5 regulates homotypic endosome fusion and incoming vesicle traffic [6,7,29,37]. The Rab4 domains, which are also present in early endosomes, organize recycling to the plasma membrane [8,9,38]. In addition, early endosomes are known to have domains containing Arf1 and COP I, caveolin-1, clathrin, and Rab11 [12,37,38,48–50].

As cargo, we made use of labeled SFV, which we could easily follow in live cells during its journey through the endosomal system. After binding to proteoglycans and internalization via coated pits, SFV particles are known to pass rapidly from early endosomes to late endosomes and lysosomes [22,23,26,51–53]. Triggered by the mildly acidic pH (<6.2.) in early endosomes, membrane fusion is induced by the E1 glycoprotein, and the capsid is released into the cytosol, where the viral RNA is rapidly uncoated [22,54,55]. After fusion with the endosomal membrane the viral glycoproteins stay together as a patch [35], and are transported to lysosomes to be degraded together with unfused virus particles [56].

The most important new observation presented in this work came from following sorting of the virus from early Rab5- and EEA1-positive endosomes to late Rab7-positive, Rab5-negative endosomes. Neutralization using NH_4_Cl showed that the infective fusion reactions occurred when the virus was still in Rab5-positive endosomes devoid of Rab7. Shortly thereafter, a large fraction of the virus-containing early endosomes became positive for Rab7. In video recordings of live cells, these Rab7-enriched domains could be seen to detach with the labeled virus from the rest of the endosome, leaving no detectable Rab7 behind. The Rab7-positive vesicles were then seen to carry the virus away along microtubules. Occasionally, fusion of such vesicles with larger Rab7-containing organelles could be seen (Videos S13 and S14). Co-immunostaining of EEA1 and Rab7 showed that Rab7 occurred in about one out of ten early endosomes, also in cells that did not contain any virus and did not express any fluorescent-protein-tagged proteins (Figure S5C).

**Figure 8:**
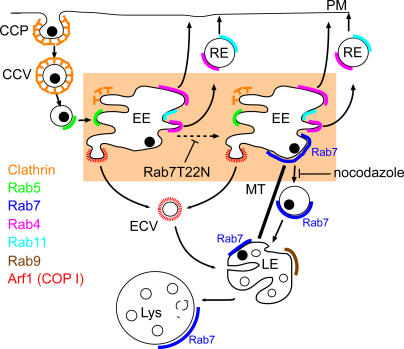
Schematic Representation of the SFV Transport Pathway from the Plasma Membrane to Lysosomes After uncoating of clathrin, the primary endocytic vesicles deliver the virus to Rab5-positive early endosomes, which contain Rab5 and Arf1 domains but do not contain Rab7. Rab7 is then recruited to these endosomes, and it forms additional domains (blue) that exclude the other early endosome-associated small GTPases. The virus moves into the Rab7 domain, and when this domain leaves the endosome as a Rab7-containing transport vesicle, the virus also leaves the Rab5-positive organelle. The Rab7-containing carrier moves along microtubules and fuses with other carriers or late endosomes. Also shown is an alternative route from the early to the late endosome, involving Arf1 and the ECV, which is not used by SFV. CCP, clathrin-coated pit; CCV, clathrin-coated vesicle; EE, early endosome; MT, microtubule; RE, recycling endosome; LE, late endosome; Lys, lysosome.

On the basis of these results, we propose that Rab7 can associate transiently with early endosomes. Since we did not observe arrival of GFP-Rab7 in vesicles, we assume that it binds directly from the cytosol. When abundant enough, it forms an endosomal domain that excludes Rab5, Rab4, Arf1, and transferrin. SFV moves selectively into this domain. Given the inhibitory effect of Rab7T22N, we further propose that the formation and/or detachment of these domains as independent carrier vesicles is Rab7-dependent. The mechanism of carrier vesicle formation appears to be independent of Arf1, and unlike Arf1-dependent formation of ECVs it is not influenced by endosomal pH. The final separation of the Rab7- and Rab5-containing elements is accelerated by transport of the Rab7-containing vesicles along microtubules. Our model is shown schematically in Figure 8. In brief, we propose that the virus uses a Rab7-regulated pathway from early to late endosomes, and that Rab7 domains in early endosomes are involved both in the sorting of the virus and in the formation of a transport vesicle.

Rab7 has been shown to be enriched in late endosomes in mammalian cells and yeast, and it is commonly thought to play an important role in fusion and transport of cargo from late endosomes to lysosomes [18–21,57]. However, a role has also been suggested in the early to late endosome transport step [14–16,57,58]. This is because overexpression of Rab7 has been shown to accelerate transfer of some cargo molecules to late endosomes and lysosomes [58,59]. Furthermore, transport of mannose 6-phosphate receptor (CI-MPR), CXCR2, cathepsin D, VSV G-protein, SV5 hemagglutinin-neuraminidase, and the LDL receptor are, like SFV, blocked at the level of early endosomes after expression of dominant negative Rab7 [14–16]. That Rab7 is involved in the transfer of other viruses is suggested by the report that influenza A virus, which fuses at the level of late endosomes, does not infect cells expressing the constitutively inactive Rab7 mutant protein [44].

Co-localization of Rab5 and Rab7 has not been observed in most localization studies [19,29]. However, consistent with our observations, partial co-localization was recently reported in HEK 293 cells [58]. One reason why the co-localization may have been missed in other studies is that only a rather small fraction (approximately 14%) of endosomes have both Rabs at any given time, and when they do, Rab7 and Rab5 (and its effector EEA1) occur in distinct domains. That the proteins are, in fact, part of the same structure is particularly apparent in video recordings where the Rab5- and Rab7-positive domains can be observed to move together as part of a common organelle. The separation of the various GTPases into distinct parts in an early endosome was not only visible when they were expressed as fluorescent-protein-tagged proteins but also when viewed in fixed control cells after immunofluorescence staining.

Since our results indicate that Rab7 is directly involved in the formation of late-endosome-bound carriers in early endosomes, one must ask what the function of the Arf1 domains is. These were clearly visible in about every third Rab5-positive early endosome. Do they induce ECVs for a parallel pathway to late endosomes, or do they serve some other function? Whereas Rab7-positive vesicles were frequently seen to depart from Rab5- and Rab4-positive endosomes, we did not observe detachment of Arf1-positive carriers corresponding to the description of ECVs. Evidence for the ECV pathway and its sensitivity to elevated lumenal pH has been obtained by in vitro experiments using purified endosome fractions [12,13]. However, it is possible that more than one pathway connects early and late endosomes, and that degradation of SFV makes use of the Rab7 pathway. Whether the recruitment of Rab7 is part of an automatic process of early endosome maturation or whether it depends on the presence of specific cargo remains to be determined. Clearly, the virus as such was not required because association of Rab7 and shedding of Rab7 vesicles from early endosomes could be readily observed also in the absence of SFV.

It will be important in future work to follow the fate of other cargo molecules in live cells, and determine whether there are indeed multiple pathways and, if so, how they relate to the Rab7 pathway. The identification of upstream regulatory elements and effectors of Rab7 must also become a priority. Currently, only a few Rab7-interacting factors are known, and none of them in early endosomes. The association of endosomes and carrier vesicles with microtubules also deserves close attention because microtubules and microtubule motors may play a role not only in the transport of vesicles but also in their formation or detachment.

## Materials and Methods

### Cells, virus, antibodies, and reagents

Vero cells were maintained in MEM (plus Earle's plus GlutaMAXI) supplemented with 10% fetal calf serum and nonessential amino acids (Gibco BRL, San Diego, California, United States). During live microscopy 20 mM Hepes (pH 7.4) was added to the medium to render the medium CO_2_-independent. A prototype tissue-culture-adapted strain of SFV was grown in BHK-21 cells and purified as described [26,60]. The virus was either fluorescently labeled immediately, or stored at −80 °C. Plaque assays were performed as described [61].

Monoclonal antibodies against human EEA1 were purchased from Transduction Laboratories (Lexington, Kentucky, United States). Rabbit polyclonal anti-Rab7 was kindly provided by Marino Zerial [36]; mouse monoclonal IgG anti-LAMP-1 (H4A3) was from Santa Cruz Biotechnology (Santa Cruz, California, United States); polyclonal antisera against E1 and E2 were raised in rabbit [62]. AF594-X and FITC-X secondary antibodies coupled to a fluorophore, and Dextran–Texas Red were from Molecular Probes (Leiden, the Netherlands). Cy5-X was from Amersham Biosciences (Little Chalfont, England), and nocodazole was from Sigma (Steinheim, Germany). All other reagents were from Sigma.

### Transfection

Cells were transfected using Nucleofactor by Amaxa (Köln, Germany) according to the following programs: Vero cells, Kit V, program A24; CV-1 cells, Kit V, program A24; and HeLa cells, Kit R, program T20. Briefly, 1 × 10^6^ Vero cells were pelleted and resuspended in 100 μl of solution V or R, and electroporated with 1–2.5 μg of DNA. The electroporated cells were resuspended in 350 μl of complete medium. Of this solution, 100 μl was seeded on one 18-mm coverslip and incubated for at least 5 h, normally overnight (15 h), at 37 °C and 5% CO_2_.

### Preparation of fluorophore-labeled SFV

Fluorescent labeling of SFV was essentially done as described [26]. Purified virus was dialyzed in 0.1 M carbonate buffer (pH 8.3), and treated for 1 h at room temperature with the different fluorophores (Cy5, FITC, or AF594) using a 1:4 molar excess of fluorophore over E1 and E2. The fluorophores react with free amines, resulting in a stable carboxamide bond with the viral proteins. The labeled virus was purified again on a 5%–60% sucrose gradient in TN buffer (50 mM Tris [pH 7.4], 100 mM NaCl). The extracted virus band was stored at 4 °C.

### Fluorescent fusion proteins

pmRFP vector was kindly provided by Roger Y. Tsien [63]. The RFP gene was cloned into the pEGFP-C3 vector (Clonetech, Palo Alto, California, United States). pmRFP-Rab5a was cloned using the restriction enzymes HindIII and PstI. pmRFP-Rab7 was constructed by PCR amplification of the Rab7 DNA from pEGFP-Rab7 using the primers 5′- CCCGATCTCGAGATGACCTCTAGG and 5′- CCCAAGCTTATCGATTTAACAACTGC, followed by cloning of the XhoI-HindIII fragment from the PCR product into pmRFP-C3 expression vector. pEGFP-Rab5 was kindly provided by M. Zerial. pEGFP-Rab7 was kindly provided by J. Gruenberg. Arf1-GFP was kindly provided by J. Lippincott-Schwartz.

### Microscopy

For live microscopy, transfected cells were seeded on 18-mm glass coverslips and before analysis transferred to custom-built metal microscope-slide chambers (Anton Lehmann, Workshop Biochemistry, Swiss Federal Institute of Technology, Zurich, Switzerland) in medium plus 20 mM Hepes (pH 7.4). The chamber was mounted on a heated stage and analyzed at 37 °C using wide field or confocal microscopy. For wide field microscopy, cells were analyzed using a Zeiss (Oberkochen, Germany) Axiovert microscope equipped with a 100× plan-apochromat lens NA 1.40. Images were collected using a cooled charge-coupled-device camera (ORCA-ER, Hamamatsu, Hamamatsu City, Japan), and the software used was OpenLab. Alternatively, an Olympus (Hamburg, Germany) IX71microscope was used with a PlanApo 60× NA 1.45 oil immersion objective and a polychrome II monochromator (TILL Photonics, Martinsried, Germany) with the TILLvisION software, version 4.0 (TILL Photonics). For confocal microscopy the Zeiss 510Meta was used with a 100× 1.4 NA objective and with an Argon laser (458, 477, 488, 514) 30 mW, HeNe laser (543 nm) 1 mW, or HeNe laser (633 nm) 5 mW.

Images were processed using Image J (National Institutes of Health, Bethesda, Maryland, United States) and Adobe Photoshop (Adobe Systems, San Jose, California, United States).

### Immunofluorescence

Cells were fixed using medium containing 4% formaldehyde, quenched with 50 mM NH_4_Cl, permeabilized with 0.05% saponin, and then incubated with primary antibodies overnight at 4 °C. Cells were incubated with the appropriate secondary antibodies. Coverslips were mounted with ImmuMount from Thermo Shandon (Pittsburgh, Pennsylvania, United States).

### Virus binding and infection

SFV was added to cells in MEM (EAGLE) containing nonessential amino acids, 5% BSA, and 20 mM Hepes (pH 7.4) and allowed to bind for 1 h at 4 °C [26]. Still on ice, the cells were washed with ice-cold PBS to remove the unbound virus. Then the cells were shifted to 37 °C for different times to start the synchronized infection. For microscopy 1–3 μg of virus was used per coverslip.

The timing of SFV penetration was determined as previously described [26]. By adding 20 mM NH_4_Cl to cells at different times after warming, acid activation of additional incoming virus particles could be blocked. To determine the fraction of cells that had been infected, a FACS-based infection assay was used [64]. The cells were stained with anti-E1/E2 antibodies and as secondary antibodies AlexaFluor488-labeled (green) or AlexaFluor647-R-phycoerythrin-labeled goat anti-rabbit IgG (Molecular Probes) was used. Analysis was performed on a FACS-Calibur cytometer using CellQuest 3.1f software (Becton-Dickinson Immunocytometry Systems, San Jose, California, United States). More than 50,000 cells were analyzed for each sample, and at least two independent experiments were performed.

### Internalization of Dextran–Texas Red

Dextran–Texas Red (10,000 MW) was added to cells on ice for at least 30 min, then the cells were shifted to 37 °C for 10 min, followed by extensive washing with warm PBS to remove the not yet internalized Dextran–Texas Red. Cells were further incubated for 2 h at 37 °C.

### Degradation of the glycoproteins E1 and E2

After binding of SFV at a MOI of 50 and washing, the cells were moved to 37 °C. At the indicated times, cells were placed on ice and incubated for 45 min with 0.5 mg/ml Proteinase K in PBS [26]. Cells were removed, washed in PBS/1 mM PMSF (phenylmethylsulfonyl fluoride)/0.2% BSA, pelleted, and dissolved in 50 μl of 50 mM Tris (pH 8.0)/2% SDS/2 mM PMSF, followed by 10 min of heating at 99 °C and TCA precipitation. The precipitate was redissolved in sample buffer, heated, and subjected to SDS-PAGE. Immunoblotting was done with an antibody against E1/E2 [62].

### Nocodazole treatment

To disrupt microtubules, 5 μM nocodazole was added to the cells. They were incubated on ice for 30 min to support disruption and then analyzed or infected with SFV and then analyzed.

### Quantification of co-localization of different endosomal markers

To quantify the co-localization of different endosomal markers, at least 16 9-μm squares were randomly placed in the cytosol of a cell. The total number of EEA1-positive endosomes and the number of structures labeled by both EEA1 and the endosomal marker of interest within those squares were counted. The endosomes labeled by both markers were expressed as percentage of EEA1-positive compartments. For each experiment at least three cells were counted.

### Using Kinetikit/GENESIS for kinetic modeling

A simple model of coupled first-order reactions was assumed and implemented using Kinetikit/GENESIS [40] (available at http://www.ncbs.res.in/∼bhalla/kkit/index.html). Simulations were carried out on a PC running Linux. Parameters were varied until they fit the experimental data. Note that the existence of other models that comply with the requirements cannot be excluded.

## Supporting Information

Figure S1 SFV-AF594 and Immunofluorescence of E1/E2 SFV-AF594 was bound to Vero cells in the cold. The cells were fixed and immunostained with a polyclonal antibody against E1/E2. SFV-AF594 co-localizes with antibodies against E1/E2. Scale bar represents 10 μm.
(8.3 MB TIF).

Figure S2 Not All Virus Particles Are Internalized after 10 min of Internalization SFV-Cy5 was bound to Vero cells for 1 h at 4 °C, and unbound virus was washed away. After 10 min of warming to 37 °C to allow internalization, the cells were fixed and incubated with antibodies against E1/E2 (SFV glycoproteins) without permeabilizing the cells. After that the cells were permeabilized and incubated with anti-EEA1. Finally, the cells were stained with secondary antibodies to E1/E2 (green) and EEA1 (red). Many of the viruses are seen to be located on the outside of the cell, as indicated by the green and blue fluorescence. The internalized viruses are only labeled with Cy5 (blue), and most of them localize with EEA1 (red).
(9.4 MB TIF).

Figure S3 Distribution of EEA1 and Rab7 in CV-1 Cells The cells were immunostained using anti-EEA1 (green) and anti-Rab7 (red) antibodies. Arrowheads show individual endosomes positive for one of the two markers, and arrows indicate endosomes positive for two markers. Scale bar represents 10 μm.
(1.1 MB TIF).

Figure S4 Distribution of EEA1 and Rab7 in HeLa Cells The cells were immunostained using anti-EEA1 (green) and anti-Rab7 (red) antibodies. Arrowheads show individual endosomes positive for one of the two markers, and arrows indicate endosomes positive for two markers. Scale bar represents 10 μm.
(1.1 MB TIF).

Figure S5 Quantification of Co-Localization of Different Markers (A and B) Confocal microscopy of Vero cells immunostained for (A) EEA1 (green) and caveolin-1 (red), or (B) EEA1 (green) and COP II (red). Shown are examples of cells, which have been quantified.
(C) Co-localization was quantified by viewing GFP-Rab5- or EEA1-containing endosomes, and determining how many of them also contained RFP-Rab7, Rab7, caveolin-1, or COP II. Scale bars represent 10 μm.
(2.6 MB TIF).

Figure S6 Kinetic Model and Parameters We had to add a pool of viruses trapped in the EEA1-positive compartment with bidirectional transport to the “normal” EEA1-positive compartment in order to fit the model to the experimental data. The average residence times of SFV in the different compartments can be calculated with the different *k*-values and the given equation for *t*_1/2_.
(328 KB TIF).

Figure S7 SFV-Cy5 Is Sorted in CV-1 and HeLa Cells (A and C) Confocal microscopy of (A) CV-1 and (C) HeLa cells. SFV-Cy5 (at a MOI of 20) was allowed to bind to the cells for 60 min at 4°C, followed by transfer to 37 °C for different times (0, 10, 20, or 30 min) and immunolabeled with anti-EEA1 and anti-Rab7 antibodies. Shown are three viruses for each time point. Scale bars represent 10 μm. At 0 min, SFV do not co-localize with any endosomal marker. At 10 min, SFV are localized in EEA1-positive endosomes. At 20 min, SFV are localized in EEA1- and Rab7-positive endosomes. At 30 min, SFV are localized in Rab7-positive endosomes.
(B and D) Selected images obtained from a time series approximately 20 min after warming. CV-1 (B) and HeLa (D) cells were transfected with GFP-Rab5 and RFP-Rab7, and SFV-Cy5 was added. SFV-Cy5 is sorted away from Rab5-positive endosomes in a Rab7-positive vesicle. Scale bars represent 2 μm.
(4.7 MB TIF).

Figure S8 SFV-Cy5 Is Sorted with RFP-Rab7 from Transferrin-AlexaFluor488 and Rab5 Selected images, obtained from time series recorded after approximately 20 min of warming. Vero cells were transfected with (A) RFP-Rab5 or (B) RFP-Rab7, loaded for 1 h with transferrin-AlexaFluor488, and SFV-Cy5 was added. SFV-Cy5 is sorted away from transferrin- and Rab5-positive endosomes in a Rab7-positive vesicle. Scale bars represent 2.5 μm.
(487 KB TIF).

Video S1 GFP-Rab5 and RFP-Rab7 Are Localized in the Same Mobile Endosome Dual-color live fluorescence microscopy experiment recorded in Vero cells expressing GFP-Rab5 and RFP-Rab7. Recording, 0.02 Hz; playback, 2 Hz. Scale bar represents 2.5 μm. Video was recorded at 37 °C with the Zeiss 510Meta confocal microscope.
(920 MB MOV).

Video S2 GFP-Rab5 and RFP-Rab7 Are Localized in the Same Mobile Endosome Dual-color live fluorescence microscopy experiment recorded in Vero cells expressing GFP-Rab5 and RFP-Rab7. Recording, 0.02 Hz; playback, 2 Hz. Scale bar represents 2.5 μm. Video was recorded at 37 °C with the Zeiss 510Meta confocal microscope.
(2.1 MB MOV).

Video S3 RFP-Rab7 Leaves a GFP-Rab5 Endosome Dual-color live fluorescence microscopy experiment recorded in Vero cells expressing RFP-Rab5 and GFP-Rab7. Recording, 0.17 Hz; playback, 2 Hz. Scale bar represents 5 μm. Video was recorded at 37 °C with the Zeiss 510Meta confocal microscope.
(839 KB MOV).

Video S4 SFV-AF594 and CFP-Rab7 Leave a YFP-Rab5-Positive Endosome Triple-color live fluorescence microscopy experiment recorded in Vero cells expressing YFP-Rab5 and CFP-Rab7 and infected with SFV-AF594. Recorded approximately 40 min after SFV addition. False colored: YFP-Rab5 (green), CFP-Rab7 (red), and SFV-AF594 (blue). Recording, 0.32 Hz; playback, 2 Hz. Scale bar represents 2.5 μm. Video was recorded with a Zeiss Axiovert microscope.
(884 KB MOV).

Video S5 SFV-Cy5 and RFP-Rab7 Leave a GFP-Rab5-Positive Endosome Triple-color live fluorescence microscopy experiment recorded in Vero cells expressing GFP-Rab5 and RFP-Rab7 and infected with SFV-Cy5. Recorded approximately 20 min after SFV addition. Recording, 0.17 Hz; playback, 2 Hz. Scale bar represents 10 μm. Video was recorded at 37 °C with the Zeiss 510Meta confocal microscope.
(3.6 MB MOV).

Video S6 SFV-Cy5 Leaves an Early Endosome Positive for GFP-Rab4 and RFP-Rab5 Triple-color live fluorescence microscopy experiment recorded in Vero cells expressing GFP-Rab4 and RFP-Rab5 and infected with SFV-Cy5. Recorded approximately 20 min after SFV addition. Recording, 0.17 Hz; playback, 2 Hz. Scale bar represents 10 μm. Video was recorded at 37 °C with the Zeiss 510Meta confocal microscope.
(1.5 MB MOV).

Video S7 SFV-Cy5 and RFP-Rab7 Leave a GFP-Rab4-Positive Endosome Triple-color live fluorescence microscopy experiment recorded in Vero cells expressing GFP-Rab4 and RFP-Rab7 and infected with SFV-Cy5. Recorded approximately 20 min after SFV addition. Recording, 0.24 Hz; playback, 2 Hz. Scale bar represents 10 μm. Video was recorded with an Olympus IX71 microscope.
(2.7 MB MOV).

Video S8 SFV-Cy5 and RFP-Rab7 Leave an Early Endosome Positive for Transferrin-AlexaFluor488 Triple-color live fluorescence microscopy experiment recorded in Vero cells expressing RFP-Rab5, loaded with transferrin-AlexaFluor488, and infected with SFV-Cy5. Recorded approximately 20 min after SFV addition. Recording, 0.17 Hz; playback, 2 Hz. Scale bar represents 2 μm. Video was recorded at 37 °C with the Zeiss 510Meta confocal microscope.
(204 KB MOV).

Video S9 SFV-Cy5 Leaves an Early Endosome Positive for Transferrin-AlexaFluor488 and RFP-Rab7 Triple-color live fluorescence microscopy experiment recorded in Vero cells expressing RFP-Rab7, loaded with transferrin-AlexaFluor488, and infected with SFV-Cy5. Recorded approximately 20 min after SFV addition. Recording, 0.17 Hz; playback, 2 Hz. Scale bar represents 2.5 μm. Video was recorded at 37 °C with the Zeiss 510Meta confocal microscope.
(272 KB MOV).

Video S10 SFV-Cy5 Leaves an Early Endosome Positive for Arf1-GFP and RFP-Rab5 Triple-color live fluorescence microscopy experiment recorded in Vero cells expressing Arf1-GFP and RFP-Rab5 and infected with SFV-Cy5. Recorded approximately 20 min after SFV addition. Recording, 0.09 Hz; playback, 2 Hz. Scale bar represents 10 μm. Video was recorded at 37 °C with the Zeiss 510Meta confocal microscope.
(1.7 MB MOV).

Video S11 SFV-Cy5 and RFP-Rab7 Leave an Arf1-GFP-Positive Endosome Triple-color live fluorescence microscopy experiment recorded in Vero cells expressing Arf1-GFP and RFP-Rab7 and infected with SFV-Cy5. Recorded approximately 20 min after SFV addition. Recording, 0.17 Hz; playback, 2 Hz. Scale bar represents 5 μm. Video was recorded at 37 °C with the Zeiss 510Meta confocal microscope.
(1 MB MOV).

Video S12 GFP-Rab7- and RFP-Rab5-Positive Endosomes Move along Microtubules (tub-YFP) Dual-color live fluorescence microscopy experiment recorded in Vero cells expressing tub-YFP, RFP-Rab5, and GFP-Rab7. Recording, 2 Hz; playback, 6 Hz. Scale bar represents 10 μm. Video was recorded at 37 °C with the Olympus IX71 microscope.
(8.2 MB KB MOV).

Video S13 Late Endosomes Labeled with CFP-Rab7 and Filled with SFV-AF594 Fuse with Other Late Endosomes Triple-color live fluorescence microscopy experiment recorded in Vero cells expressing YFP-Rab5 and CFP-Rab7 and infected with SFV-AF594. Recorded approximately 40 min after SFV addition. False colored: YFP-Rab5 (green), CFP-Rab7 (red), and SFV-AF594 (blue). Recording, 0.32 Hz; playback, 6 Hz. Scale bar represents 2.5 μm. Video was recorded with a Zeiss Axiovert microscope.
(1.8 MB MOV).

Video S14 Fusion of Late Endosomes Labeled with RFP-Rab7 and Filled with SFV-Cy5 Dual-color live fluorescence microscopy experiment recorded in Vero cells expressing RFP-Rab5 and infected with SFV-Cy5. Recorded approximately 30 min after SFV addition. Recording, 0.17 Hz; playback, 2 Hz. Scale bar represents 5 μm. Video was recorded at 37 °C with the Zeiss 510Meta confocal microscope.
(1.3 MB MOV).

### Accession Numbers

The Entrez Gene (http://www.ncbi.nlm.nih.gov/entrez/query.fcgi?db=gene ) accession numbers of the proteins used in this paper are Arf1 (ID 375), EEA1 (ID 8411), LAMP-1 (ID 16783), Rab4 (ID 5867), Rab5 (ID 5868), and Rab7 (ID 7879).

## Abbreviations

AF594: AlexaFluor594
CFP: cyan fluorescent protein
ECV: endosomal carrier vesicle
EEA1: early endosome antigen 1
GFP: green fluorescent protein
LAMP-1: lysosome associated membrane protein 1
MOI: multiplicity of infection
PFU: plaque forming units
RFP: red fluorescent protein
SFV: Semliki forest virus
YFP: yellow fluorescent protein

## References

[pbio-0030233-b01] Aniento F, Emans N, Griffiths G, Gruenberg J (1993). Cytoplasmic dynein-dependent vesicular transport from early to late endosomes. J Cell Biol.

[pbio-0030233-b02] Bomsel M, Parton R, Kuznetsov SA, Schroer TA, Gruenberg J (1990). Microtubule- and motor-dependent fusion in vitro between apical and basolateral endocytic vesicles from MDCK cells. Cell.

[pbio-0030233-b03] Gruenberg J, Stenmark H (2004). The biogenesis of multivesicular endosomes. Nat Rev Mol Cell Biol.

[pbio-0030233-b04] Novick P, Zerial M (1997). The diversity of Rab proteins in vesicle transport. Curr Opin Cell Biol.

[pbio-0030233-b05] Pfeffer SR (2001). Rab GTPases: Specifying and deciphering organelle identity and function. Trends Cell Biol.

[pbio-0030233-b06] Gorvel JP, Chavrier P, Zerial M, Gruenberg J (1991). rab5 controls early endosome fusion in vitro. Cell.

[pbio-0030233-b07] Bucci C, Parton RG, Mather IH, Stunnenberg H, Simons K (1992). The small GTPase rab5 functions as a regulatory factor in the early endocytic pathway. Cell.

[pbio-0030233-b08] van der Sluijs P, Hull M, Webster P, Male P, Goud B (1992). The small GTP-binding protein rab4 controls an early sorting event on the endocytic pathway. Cell.

[pbio-0030233-b09] Sheff DR, Daro EA, Hull M, Mellman I (1999). The receptor recycling pathway contains two distinct populations of early endosomes with different sorting functions. J Cell Biol.

[pbio-0030233-b10] Whitney JA, Gomez M, Sheff D, Kreis TE, Mellman I (1995). Cytoplasmic coat proteins involved in endosome function. Cell.

[pbio-0030233-b11] Gu F, Gruenberg J (1999). Biogenesis of transport intermediates in the endocytic pathway. FEBS Lett.

[pbio-0030233-b12] Gu F, Gruenberg J (2000). ARF1 regulates pH-dependent COP functions in the early endocytic pathway. J Biol Chem.

[pbio-0030233-b13] Clague MJ, Urbe S, Aniento F, Gruenberg J (1994). Vacuolar ATPase activity is required for endosomal carrier vesicle formation. J Biol Chem.

[pbio-0030233-b14] Feng Y, Press B, Wandinger-Ness A (1995). Rab 7: An important regulator of late endocytic membrane traffic. J Cell Biol.

[pbio-0030233-b15] Fan GH, Lapierre LA, Goldenring JR, Richmond A (2003). Differential regulation of CXCR2 trafficking by Rab GTPases. Blood.

[pbio-0030233-b16] Press B, Feng Y, Hoflack B, Wandinger-Ness A (1998). Mutant Rab7 causes the accumulation of cathepsin D and cation-independent mannose 6-phosphate receptor in an early endocytic compartment. J Cell Biol.

[pbio-0030233-b17] Miaczynska M, Zerial M (2002). Mosaic organization of the endocytic pathway. Exp Cell Res.

[pbio-0030233-b18] Bucci C, Thomsen P, Nicoziani P, McCarthy J, van Deurs B (2000). Rab7: A key to lysosome biogenesis. Mol Biol Cell.

[pbio-0030233-b19] Bottger G, Nagelkerken B, van der Sluijs P (1996). Rab4 and Rab7 define distinct nonoverlapping endosomal compartments. J Biol Chem.

[pbio-0030233-b20] Meresse S, Gorvel JP, Chavrier P (1995). The rab7 GTPase resides on a vesicular compartment connected to lysosomes. J Cell Sci.

[pbio-0030233-b21] Schimmoller F, Riezman H (1993). Involvement of Ypt7p, a small GTPase, in traffic from late endosome to the vacuole in yeast. J Cell Sci.

[pbio-0030233-b22] Marsh M, Helenius A (1989). Virus entry into animal cells. Adv Virus Res.

[pbio-0030233-b23] Doxsey SJ, Brodsky FM, Blank GS, Helenius A (1987). Inhibition of endocytosis by anti-clathrin antibodies. Cell.

[pbio-0030233-b24] DeTulleo L, Kirchhausen T (1998). The clathrin endocytic pathway in viral infection. EMBO J.

[pbio-0030233-b25] Mancini EJ, Clarke M, Gowen BE, Rutten T, Fuller SD (2000). Cryo-electron microscopy reveals the functional organization of an enveloped virus, Semliki Forest virus. Mol Cell.

[pbio-0030233-b26] Helenius A, Kartenbeck J, Simons K, Fries E (1980). On the entry of semliki forest virus into BHK-21 cells. J Cell Biol.

[pbio-0030233-b27] Christoforidis S, McBride HM, Burgoyne RD, Zerial M (1999). The Rab5 effector EEA1 is a core component of endosome docking. Nature.

[pbio-0030233-b28] Simonsen A, Lippe R, Christoforidis S, Gaullier JM, Brech A (1998). EEA1 links PI(3)K function to Rab5 regulation of endosome fusion. Nature.

[pbio-0030233-b29] Chavrier P, Parton RG, Hauri HP, Simons K, Zerial M (1990). Localization of low molecular weight GTP binding proteins to exocytic and endocytic compartments. Cell.

[pbio-0030233-b30] Carlsson SR, Roth J, Piller F, Fukuda M (1988). Isolation and characterization of human lysosomal membrane glycoproteins, h-lamp-1 and h-lamp-2. Major sialoglycoproteins carrying polylactosaminoglycan. J Biol Chem.

[pbio-0030233-b31] Kornfeld S, Mellman I (1989). The biogenesis of lysosomes. Annu Rev Cell Biol.

[pbio-0030233-b32] Ohkuma S, Poole B (1978). Fluorescence probe measurement of the intralysosomal pH in living cells and the perturbation of pH by various agents. Proc Natl Acad Sci U S A.

[pbio-0030233-b33] Helenius A, Marsh M (1982). Endocytosis of enveloped animal viruses. Ciba Found Symp.

[pbio-0030233-b34] Marsh M, Helenius A (1980). Adsorptive endocytosis of Semliki Forest virus. J Mol Biol.

[pbio-0030233-b35] White J, Helenius A (1980). pH-dependent fusion between the Semliki Forest virus membrane and liposomes. Proc Natl Acad Sci U S A.

[pbio-0030233-b36] Nielsen E, Severin F, Backer JM, Hyman AA, Zerial M (1999). Rab5 regulates motility of early endosomes on microtubules. Nat Cell Biol.

[pbio-0030233-b37] Pelkmans L, Burli T, Zerial M, Helenius A (2004). Caveolin-stabilized membrane domains as multifunctional transport and sorting devices in endocytic membrane traffic. Cell.

[pbio-0030233-b38] Sonnichsen B, De Renzis S, Nielsen E, Rietdorf J, Zerial M (2000). Distinct membrane domains on endosomes in the recycling pathway visualized by multicolor imaging of Rab4, Rab5, and Rab11. J Cell Biol.

[pbio-0030233-b39] Zerial M, McBride H (2001). Rab proteins as membrane organizers. Nat Rev Mol Cell Biol.

[pbio-0030233-b40] Bhalla US, Iyengar R (1999). Emergent properties of networks of biological signaling pathways. Science.

[pbio-0030233-b41] Bananis E, Murray JW, Stockert RJ, Satir P, Wolkoff AW (2000). Microtubule and motor-dependent endocytic vesicle sorting in vitro. J Cell Biol.

[pbio-0030233-b42] Herman B, Albertini DF (1984). A time-lapse video image intensification analysis of cytoplasmic organelle movements during endosome translocation. J Cell Biol.

[pbio-0030233-b43] Matteoni R, Kreis TE (1987). Translocation and clustering of endosomes and lysosomes depends on microtubules. J Cell Biol.

[pbio-0030233-b44] Sieczkarski SB, Whittaker GR (2003). Differential requirements of Rab5 and Rab7 for endocytosis of influenza and other enveloped viruses. Traffic.

[pbio-0030233-b45] Helenius A, Mellman I, Wall D, Hubbard A (1983). Endosomes. Trends in Biochem Sci.

[pbio-0030233-b46] Gruenberg J (2001). The endocytic pathway: A mosaic of domains. Nat Rev Mol Cell Biol.

[pbio-0030233-b47] de Renzis S, Sonnichsen B, Zerial M (2002). Divalent Rab effectors regulate the sub-compartmental organization and sorting of early endosomes. Nat Cell Biol.

[pbio-0030233-b48] Raiborg C, Bache KG, Gillooly DJ, Madshus IH, Stang E (2002). Hrs sorts ubiquitinated proteins into clathrin-coated microdomains of early endosomes. Nat Cell Biol.

[pbio-0030233-b49] Sachse M, Urbe S, Oorschot V, Strous GJ, Klumperman J (2002). Bilayered clathrin coats on endosomal vacuoles are involved in protein sorting toward lysosomes. Mol Biol Cell.

[pbio-0030233-b50] Miaczynska M, Pelkmans L, Zerial M (2004). Not just a sink: Endosomes in control of signal transduction. Curr Opin Cell Biol.

[pbio-0030233-b51] Klimstra WB, Ryman KD, Johnston RE (1998). Adaptation of Sindbis virus to BHK cells selects for use of heparan sulfate as an attachment receptor. J Virol.

[pbio-0030233-b52] Byrnes AP, Griffin DE (1998). Binding of Sindbis virus to cell surface heparan sulfate. J Virol.

[pbio-0030233-b53] Smit JM, Waarts BL, Kimata K, Klimstra WB, Bittman R (2002). Adaptation of alphaviruses to heparan sulfate: Interaction of Sindbis and Semliki forest viruses with liposomes containing lipid-conjugated heparin. J Virol.

[pbio-0030233-b54] Kielian M (1995). Membrane fusion and the alphavirus life cycle. Adv Virus Res.

[pbio-0030233-b55] Wahlberg JM, Bron R, Wilschut J, Garoff H (1992). Membrane fusion of Semliki Forest virus involves homotrimers of the fusion protein. J Virol.

[pbio-0030233-b56] Marsh M, Bolzau E, Helenius A (1983). Penetration of Semliki Forest virus from acidic prelysosomal vacuoles. Cell.

[pbio-0030233-b57] Papini E, Satin B, Bucci C, de Bernard M, Telford JL (1997). The small GTP binding protein rab7 is essential for cellular vacuolation induced by *Helicobacter pylori* cytotoxin. EMBO J.

[pbio-0030233-b58] Dale LB, Seachrist JL, Babwah AV, Ferguson SS (2004). Regulation of angiotensin II type 1A receptor intracellular retention, degradation, and recycling by Rab5, Rab7, and Rab11 GTPases. J Biol Chem.

[pbio-0030233-b59] Mukhopadhyay A, Barbieri AM, Funato K, Roberts R, Stahl PD (1997). Sequential actions of Rab5 and Rab7 regulate endocytosis in the *Xenopus* oocyte. J Cell Biol.

[pbio-0030233-b60] Kaariainen L, Simons K, von Bonsdorff CH (1969). Studies in subviral components of Semliki Forest virus. Ann Med Exp Biol Fenn.

[pbio-0030233-b61] Kielian M, Keranen S, Kaariainen L, Helenius A (1984). Membrane fusion mutants of Semliki Forest virus. J Cell Biol.

[pbio-0030233-b62] Singh I, Helenius A (1992). Role of ribosomes in Semliki Forest virus nucleocapsid uncoating. J Virol.

[pbio-0030233-b63] Campbell RE, Tour O, Palmer AE, Steinbach PA, Baird GS (2002). A monomeric red fluorescent protein. Proc Natl Acad Sci U S A.

[pbio-0030233-b64] Sieczkarski SB, Whittaker GR (2002). Dissecting virus entry via endocytosis. J Gen Virol.

